# Additive‐Free Formic Acid Dehydrogenation Catalyzed by a Cp*Ir Complex with Pyridyl‐Pyrazole Ligand: Long‐Term Hydrogen Generation and Impurity Effects

**DOI:** 10.1002/open.202600003

**Published:** 2026-02-17

**Authors:** Naoya Onishi, Yuichiro Himeda

**Affiliations:** ^1^ Global Zero Emission Research Center National Institute of Advanced Industrial Science and Technology Tsukuba West Japan

**Keywords:** formic acid, hydrogen evolutionl, iridium catalyst

## Abstract

Hydrogen generation from formic acid (FA), as one of the most promising hydrogen carriers, has attracted significant attention due to the growing demand for renewable energy carriers. FA dehydrogenation (HCOOH → H_2_ + CO_2_) offers an efficient and environmentally friendly pathway but remains challenging, particularly regarding catalyst durability. While numerous studies have focused on enhancing catalytic activity, this article emphasizes catalyst design for improved durability, leading to the development of a novel catalyst that achieves both high activity and long‐term stability. Ir complexes with pyridyl‐pyrazole ligands with electron‐donating substituents on the pyridine and pyrazole moieties completed the reaction without apparent degradation under reflux conditions and exhibited excellent durability. Moreover, FA dehydrogenation using this catalyst was sustained over an extended period by continuously pumping a formic acid solution, generating 3.3 m^3^ of gases over 43 days. The effects of impurities on the catalytic reaction were also examined, revealing that NaCl significantly inhibited the reaction. These findings provide valuable insights into the practical application of hydrogen generation through FA dehydrogenation.

## Graphical abstract

Novel Cp*Ir catalysts bearing pyridyl‐pyrazole ligands with electron‐donating groups exhibited both high catalytic activity and durability for FADH. The catalyst produced 3.3 m^3^ of gas and maintained catalytic activity for 43 days.

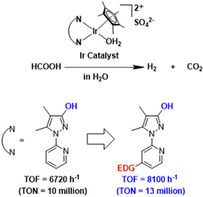

## 1 Introduction

Due to the deteriorating global environment, an immediate shift to decarbonized and sustainable energy sources is essential. Among various renewable options, hydrogen has gained increasing attention in recent years because of its high energy density and environmental compatibility [1]. Although the gravimetric energy density of H_2_ is exceptionally high, its volumetric energy density is low under ambient conditions, resulting in higher costs for storage and transportation. Consequently, the development of hydrogen carriers for efficient storage and transport has become an active area of research, with ammonia [2] and organic hydrides [3] attracting particular interest. In addition, high‐pressure hydrogen must be produced at hydrogen stations using environmentally benign methods to avoid the economic losses associated with pressurization.

Formic acid (FA) is considered one of the most promising hydrogen carriers because it is a liquid containing 4.4% hydrogen by weight and has low toxicity to both humans and the environment [4, 5, 6, 7, 8, 9, 10, 11]. Another advantage is that FA can be synthesized from carbon dioxide, an inexpensive and abundant greenhouse gas (CO_2_ + H_2_ → HCOOH). Furthermore, the free energy required for interconversion with carbon dioxide in aqueous solution is 4 kJ/mol, which is much lower than that of other hydrogen carriers. Therefore, if FA can be employed as a hydrogen carrier, the energy loss associated with material conversion during hydrogen storage and release can be significantly reduced. In addition, unlike other hydrogen carriers, FA dehydrogenation (FADH) proceeds even under pressure, enabling the supply of compressed hydrogen [12, 13, 14]. To exploit the excellent properties of FA as a hydrogen carrier, the development of high‐performance catalysts capable of efficiently generating hydrogen from FADH has been vigorously pursued.

**SCHEME 1:**
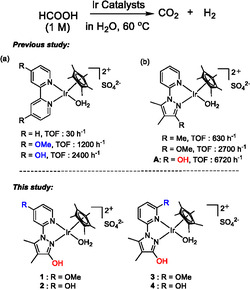
FADH with Ir catalysts.

Since Laurenczy et al. and Beller et al. independently reported in 2008 that Ru catalysts are effective for FADH [15, 16], several transition‐metal catalysts, including Fe [17, 18, 19, 20, 21, 22, 23, 24], Ru [25, 26, 27, 28, 29, 30], and Ir [31, 32, 33, 34, 35, 36, 37, 38, 39, 40, 41, 42, 43, 44, 45, 46, 47, 48], have been studied. We reported that half‐sandwich complexes with *N*,*N*′‐bidentate ligands such as 2,2′‐bipyridine are active for hydrogen production from FA in aqueous solution [49]. It has also been reported that introducing an electron‐donating group onto the bidentate ligand in Cp*Ir complexes improves catalytic activity (Scheme 1a) [50, 51]. Catalytic activity can further be enhanced by employing ligands such as imidazole [52] or imidazoline [53] instead of a pyridine ring. Improved activity through the introduction of electron‐donating substituents has also been observed with pyrazole‐based catalysts (Scheme 1b) [54]. When catalysts with various substituents (Me, OMe, or OH group) at the pyrazole moiety were examined, catalyst **A** (Scheme 1b, R=OH) bearing the OH group exhibited particularly high activity. In addition, **A** withstood prolonged reaction times, operating for up to 35 days. Both activity and durability are essential for the practical application of such catalysts. Because improvements in both catalytic activity and durability for FADH are required, new high‐performance catalysts can be developed by building on these insights. Recently, Nielsen et al. reported a novel Ru catalyst bearing a PNP‐pincer ligand for FADH with a TON (Turnover Number) of 18 million [55]. However, their system required an ionic liquid as a solvent. By contrast, our system employs only water as a solvent and operates under additive‐free conditions, making it environmentally friendly.

In addition, the effect of impurities in FA must be considered for practical applications. In the future, it is hoped that formic acid will be produced from CO_2_ or biomass rather than fossil resources. FA supplied by the electroreduction of CO_2_ is expected to contain impurities, such as KCl, which is used as an electrolyte [56]. It is known that FA obtained from biomass contains various residues in the process of its conversion [57].

Herein, we report the introduction of electron‐donating substituents into the pyridine ring of **A** (i.e., complexes **1**‐**4** in Scheme 1), which resulted in significantly higher activity and durability for the water‐soluble complexes **1** and **2**. For practical application, we further investigated the long‐term durability of **1** and examined the effect of impurities in FA.

## 2 Experimental Section

### 2.1 General Analytical and Experimental Information

Unless otherwise noted, materials were purchased from commercial suppliers and used without further purification. All manipulations were carried out under an inert atmosphere using standard Schlenk techniques or in a glovebox, and all aqueous solutions were degassed prior to use. ^1^H NMR (Nuclear Magnetic Resonance) and ^13^C{^1^H} NMR spectra were recorded on Bruker Avance 400 and 500 spectrometers. Elemental analyses were performed by a CE Instruments EA1110 elemental analyzer. An Orion 3‐Star pH meter with a glass electrode was used to measure pH values after calibration with standard buffer solutions. ESI‐MS (Electrospray Ionization Mass Spectrometry) data were collected on a Shimadzu LCMS‐2020. Formate concentrations were monitored by an HPLC (High Performance Liquid Chromatography) on an anion‐exclusion column (Tosoh TSKgel SCX(H+)) using an aqueous H_3_PO_4_ solution (20 mM) as an eluent and a UV detector (*λ *= 210 nm). Water used in the reactions was obtained from a Simplicity water purification system. Unless specifically noted, all reagents were purchased commercially without further purification. [Cp*Ir(OH_2_)_3_][SO_4_] was synthesized according to a previous report.

### 2.2 General Procedure for FA Dehydrogenation

A freshly prepared 10 mM aqueous solution of catalyst (100 μL) was added to a deaerated aqueous HCO_2_H (FA)/HCO_2_Na (SF) solution, and the mixture was stirred at the desired temperature. The volume of released gases was determined by a wet gas meter. The TOF (Turnover Frequency) was determined according to the released gases. The average TOF of the initial 10 min was adopted as the initial TOF. After the reaction was completed, the residual FA in the solution was quantified with HPLC. The TON was calculated based on the catalyst loading and concentration of residual FA or formate.

***Note***: The solubility of catalyst **2** in neutral water is relatively poor. Therefore, catalyst **2** was added to a FA solution after being dissolved in an alkaline solution with NaOH (pH > 12).

## 3 Results and Discussion

### 3.1 Catalytic Activity for FADH

As shown in Scheme 1a, the introduction of electron‐donating substituents (OMe, OH) into the bipyridine ligand improves the catalytic activity of FADH [50]. It has also been shown that the introduction of electron‐donating groups (OMe, OH) on the pyrazole moiety of the pyridyl‐pyrazole ligand improves the catalytic activity of FADH (Scheme 1b) [54]. Based on these findings, catalysts **1**–**4** were prepared, each bearing a pyridyl‐pyrazole ligand with an electron‐donating substituent. Complexes **1–4** were newly synthesized and characterized by NMR spectroscopy and elemental analysis (details in ESI). Their catalytic activities for FADH were evaluated in aqueous FA solution, as summarized in Table 1 (conditions: [FA] = 1.0 M, [cat] = 100 μM, 10 mL, 60°C). The initial TOFs obtained with **1** and **2**, which possess electron‐donating substituents at the 4‐position of the pyridine ring, were higher than those of the previously reported analog **A** (Entries 1 and 2 vs. 5). In contrast, introducing OH or OMe substituents at the 6‐position of the pyridine ring did not improve catalytic activity (Entries 3 and 4), likely because steric hindrance restricted substrate access to the metal center.

**TABLE 1:** FADH results with catalysts **1–4** and **A**.^a^

Entry	Cat/μM	FA conc.	Temp.	TOF^b^, h^−1^
1	**1**/100	1 M	60°C	8210
2	**2**/100	1 M	60°C	8710
3	**3**/100	1 M	60°C	4500
4	**4**/100	1 M	60°C	4600
5^c^	**A**/100	1 M	60°C	6760
6	**1**/10	8 M	reflux	156,000
7	**2**/10	8 M	reflux	161,000
8	**3**/10	8 M	reflux	66,000
9	**4**/10	8 M	reflux	59,500
10^c^	**A**/10	8 M	reflux	111,000

a Reaction was performed in deaerated aqueous FA solution.

b Initial TOF was measured after 10 min.

c Ref. [54].

**FIGURE 1:**
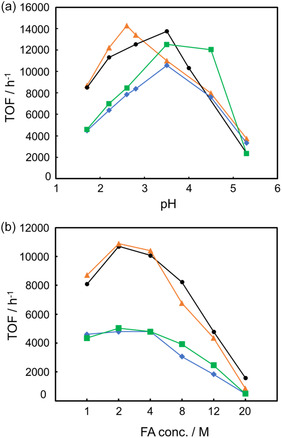
(a) pH dependence of TOF with catalysts **1–4** in a FA/HCO_2_Na solution (10 mL; 1 M) at 60°C. The solution pH was adjusted by varying the ratio of FA and HCO_2_Na while keeping their total concentration constant (1 M). (b) Initial TOF (average rate over the first 10 min) plotted against FA concentration of the reaction solution with 0.1 mM of catalysts **1–4** at 60°C. •: catalyst **1**, 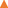: catalyst **2**, 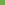: catalyst **3**, 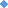: catalyst **4**.

The influence of reaction conditions, including FA concentration, temperature, and solution pH, was investigated using **1–4**. To examine pH dependence, FADH was examined over a wide range of pH values adjusted with sodium formate (HCO_2_Na). As shown in Figure 1a, the obtained TOFs were strongly pH dependent, with pseudo‐bell‐shaped profiles observed for all catalysts **1–4**. The effect of FA concentration on FADH was then evaluated. As shown in Figure 1b, the reaction rate depended on the FA concentration. At 2 and 4 M FA, **1–4** exhibited high TOFs. At 20 M FA, the initial TOFs for **1–4** were much lower, ranging from 490 to 1600 h^−1^; however, the reaction rates gradually increased during the reaction due to the decrease in FA concentration. The effect of temperature on FADH was subsequently studied using **1** and **2** (Table S1, Figure S1). As expected, the TOFs increased with temperature, approximately doubling with each 10°C rise. Analysis of Arrhenius plots yielded activation energies (*E*_a_) of 71.6 and 71.5 kJ/mol for **1** and **2**, respectively, which are lower than the 74.1 kJ/mol reported for **A** in a previous study.

### 3.2 Mechanistic Study of Catalysis

To confirm the reaction mechanism, an equal amount of sodium formate was reacted with catalyst **1**. As a result, the reaction solution turned from yellow to red immediately after HCOONa was added to the catalyst solution. Based on previous research, this is presumed to be due to the formation of an Ir—H complex. However, when the reaction solution was analyzed by NMR spectrum, no peaks attributed to the Ir—H complex were observed. This is likely due to H‐D exchange between Ir—H and D_2_O, forming Ir—D. Although no peaks attributed to Ir—H were observed, the peaks attributed to aromatic rings did change, suggesting that the reaction had progressed. When D_2_SO_4_ was added to the reaction solution, the reaction solution returned to yellow, and peaks attributed to catalyst **1** were observed. Based on these results, the reaction mechanism of catalyst **1** is thought to involve a reaction with formate ions to generate Ir—H as an active species, which then reacts with protons present in the reaction system to generate hydrogen.

### 3.3 Durability Test

**FIGURE 2:**
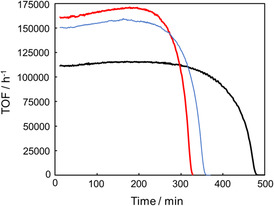
Time courses of TOFs for FADH under reflux conditions: blue line represents catalyst **1**, red line represents catalyst **2**, black line represents catalyst **A**. (Conditions: [FA] = 8 M, 100 mL, [cat] = 10 μM, reflux).

Durability is a critical factor for the practical application of catalysts, and relevant information is typically obtained through durability studies. In our recent article, we demonstrated that **A** maintained its catalytic activity under reflux conditions, achieving a TOF of more than 110,000 h^−1^ for at least the first 5 h and completing the reaction in 8 h (black line in Figure 2, Entry 10 in Table 1) [54]. Furthermore, **A** sustained its activity for 35 days at 70°C using a pump to continuously add FA, resulting in a total TON of 10 million and demonstrating exceptional durability. To further improve catalytic activity and durability, we evaluated **1** and **2**, which showed much higher catalytic activity than **3** and **4**, under reflux conditions ([FA] = 8.0 M, [cat] = 10 μM, 100 mL, reflux; Figure 2, Entries 6–10 in Table 1). No apparent decrease in activity was observed for any of the catalysts, indicating extraordinary durability (TON = 800,000).

**FIGURE 3:**
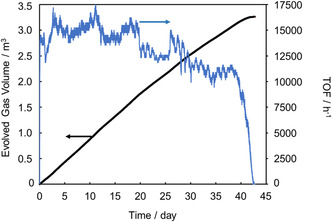
Time courses of volume of released gases (black line) and rate of released gases (blue line) in FADH with the continuous addition of FA by a pump. Conditions: [FA]_0_ = 4.0 M, 500 mL, [**1**]_0_ = 10 μM, 70°C, [FA]_add_ = 65 wt% (= 16 M), rate of FA addition = 0.085 ml/min, total additional FA amount = 66 mol.

To further compare durability, a 20 M (80 wt%) FA solution was dehydrogenated under reflux using **1** and **2**. Catalyst **1** completed the reaction without any loss of activity, whereas **2** was deactivated 5 h after the start, achieving only 10% FA conversion. Next, the durability of **1** was further evaluated during FADH with neat FA fed by a liquid feeding pump (Figure S2). The catalytic activity was maintained for nearly 100 h, generating 800 L of gas (TON = 4 million). Based on these results, **1** was identified as the most durable and its lifetime was further assessed under continuous FA addition using a pump (Figure 3; conditions: [FA]_0_ = 4.0 M, 500 mL, [**1**]_0_ = 10 µM, [FA]_add_ = 16 M, addition rate = 0.085 mL/min, at 70°C). Although the reaction rate fluctuated, likely due to difficulties in maintaining steady heating, the catalyst remained active for 43 days, producing a total TON of 13 million and 3.3 m^3^ of evolved gas volume. While some catalyst degradation may have occurred during this long‐term reaction, this result does not indicate the intrinsic lifetime limit of **1**. After 33 days, FA addition had to be stopped because the water used as a solvent in the FA solution increased the total volume, nearly causing overflow from the reaction vessel. Had the overflow been avoided or a larger‐capacity vessel employed, the catalytic activity could likely have been sustained for an even longer period.

**FIGURE 4:**
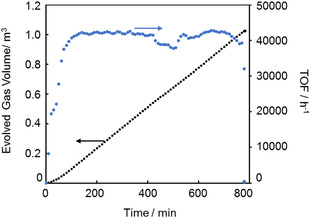
Time courses of volume of released gases (black line) and rate of released gases (blue line) in FADH for the generation of a large amount of H_2_. (Conditions: [FA] = 10 M, 2 L, [**1**] = 20 μM, bath temp. 130°C).

To produce a large volume of H_2_ in a short period, FADH was performed with a larger amount of catalyst **1** under high‐temperature conditions (Figure 4; [FA] = 10 M, 2 L, [**1**] = 20 μM (26 mg), oil bath temperature = 130°C). This setup resulted in the generation of 1 m^3^ of gas in just half a day. Because the volume of the reaction solution was large, heating was not sufficient, and the solution temperature remained at around 80°C, so the TOF was about 40,000 h^−1^, but the reaction was completed. These results indicate that the catalyst is highly durable under FADH conditions, even with high FA concentrations and elevated temperatures. Therefore, the feasibility of this catalyst for practical application can be further evaluated in a suitable pilot plant.

### 3.4 Effect of Catalyst Structure on Durability

**FIGURE 5:**
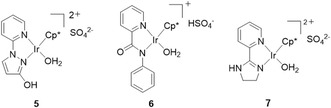
Structure of catalysts **5**–**7**.

As mentioned above, the excellent durability of catalyst **1** was demonstrated, and a comparison with other catalysts **5**–**7** (Figure 5) was also carried out. When the durability test was performed with **5** under reflux conditions ([FA] = 8.0 M, [cat] = 10 μM, 100 mL), the TOF declined from the initial stage of the reaction (Figure S3a). This suggests that the Me group on the pyrazole moiety in the ligand may introduce a rotational barrier between the pyridine and pyrazole rings. Indeed, previous studies have reported that such rotational barriers can lead to catalyst deactivation [12]. Furthermore, the durability of the catalyst was also compared with previously reported catalysts. Picolinamide catalyst **6** was more catalytically active than catalyst **1** under mild reaction conditions (TOF of 20,800 hr^−1^, conditions: [FA] = 1.0 M, 60°C) [57], but the catalyst was quickly deactivated under reflux conditions (Figure S3b). Similarly, pyridyl‐imidazoline catalyst **7** was more active than catalyst **1** under mild reaction conditions (TOF of 13,300 h^−1^, conditions: [FA] = 1.0 M, 60°C) [38], but the catalyst was gradually deactivated under reflux conditions (Figure S3c). The difference in durability between these two catalysts and the catalyst **1** is likely due to the presence or absence of aromaticity.

### 3.5 Influence of Impurities Toward FADH

**FIGURE 6:**
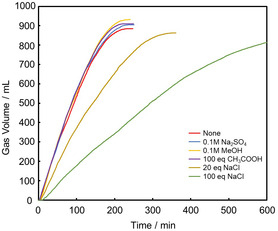
Time course plots of FA dehydrogenation catalyzed by catalyst **1** in the presence of the various impurities. Conditions: [FA] = 1.0 M, [**1**] = 50 μM, at 60°C.

Next, the effect of various impurities (MeOH, Na_2_SO_4_, CH_3_COOH, and NaCl) on catalytic activity was investigated. These four compounds were selected because MeOH can be produced by disproportionation of formic acid (3HCOOH → CH_3_OH + 2CO_2_ + H_2_O) as a side reaction of FADH. Furthermore, because industrial formic acid is sometimes produced by adding H_2_SO_4_ to sodium formate (HCOONa), the effect of Na_2_SO_4_ was also investigated. Furthermore, formic acid is produced as a by‐product in the industrial production of acetic acid, and the possibility of acetic acid being present in the formic acid purification process was considered. Finally, in the currently actively studied formic acid synthesis by CO_2_ electrolysis, NaCl is used as the electrolyte, so it may be mixed in as an impurity. Figure 6 shows the time course of volume of released gases on FADH in the presence of **1**, with or without additives such as MeOH, Na_2_SO_4_, CH_3_COOH, or NaCl. All additives except NaCl had little effect on the reaction, which is consistent with the previous report [58]. However, increasing amounts of NaCl tended to decrease the reaction rate. This suggests that Cl^−^ coordinates strongly to the metal center, hindering the exchange of Cl^−^ and HCOO^−^ required to form the reaction intermediate.

## 4 Conclusion

In summary, by modifying both coordination moieties of the pyridyl‐pyrazole ligand with electron‐donating substituents, we successfully developed highly active and durable catalysts **1** and **2**. The introduction of electron‐donating substituents on the pyridine and pyrazole moieties enhanced catalytic activity. Notably, **1** exhibited exceptional durability, showing no deactivation even under reflux conditions. Its catalytic performance was maintained during continuous FA addition for 43 days, achieving a TON of 13 million—one of the highest values reported under aqueous [59], additive‐free conditions—and generating 3.3 m^3^ of gas. Additionally, studies on the effects of impurities revealed that impurities had little effect on catalytic activity, except for Cl anions.

## Supporting Information

Additional supporting information can be found online in the Supporting Information section. **Supporting** **Fig. S1:** Arrhenius plots for FADH (a) with **1** and (b) with **2**. Reaction conditions: [FA] = 1.0 M, [cat] = 100 mM. **Supporting** **Fig.** **S2:** Time courses of volume of released gases and rate of released gases in FADH with the continuous addition of neat FA by a pump. Conditions: [FA]0 = 8 M, 50 mL, [**1**]0 = 80 mM, 70°C, rate of neat FA addition = 0.1 ml/min. **Supporting** **Fig.** **S3:** Time courses of volume of released gases and rate of released gases in FADH catalyzed by catalyst **5‐7**. Conditions: [FA] = 8 M, 100 mL, [**cat**] = 10 mM, reflux). **Supporting** **Table** **S1:** The results of FADH under various temperature conditions^a^. **Supporting Table S2**. FA dehydrogenation in the presence of impurities^a^.

## Funding

This work was supported by Japan Society for the Promotion of Science (23K04923, 23H00315), and Cabinet Office, Government of Japan.

## Conflicts of Interest

The authors declare no conflicts of interest.

## Supporting information

Supplementary Material

## Data Availability

The data that support the findings of this study are available in the supplementary material of this article.
