# Supplementary material for: Additive‐Free Formic Acid Dehydrogenation Catalyzed by a Cp*Ir Complex with Pyridyl‐Pyrazole Ligand: Long‐Term Hydrogen Generation and Impurity Effects
Source: ChemistryOpen. 2026 Feb 17;15(2):e202600003. doi: 10.1002/open.202600003 (PMC12910247; doi:10.1002/open.202600003)
Supplement: Supplementary file 1 — Supplementary Material [file OPEN-15-e202600003-s001.pdf]

Additive-Free Formic Acid Dehydrogenation Catalyzed by a Cp*Ir Complex with Pyridyl-pyrazole Ligand: Long-Term Hydrogen Generation and Impurity Effects

Naoya Onishi,[a] Yuichiro Himeda[*a]

a. Global Zero Emission Research Center, National Institute of Advanced Industrial Science and Technology, Tsukuba West, 16-1 Onogawa, Tsukuba, Ibaraki, 305-8569, Japan.

1. General Analytical and Experimental Information

Materials and Equipment: Unless otherwise noted, materials were purchased from commercial suppliers and used without further purification. All manipulations were carried out under an inert atmosphere using standard Schlenk techniques or in a glovebox, and all aqueous solutions were degassed prior to use. ^{1}H NMR and ^{13}C{^{1}H} NMR spectra were recorded on Bruker Avance 400 and 500 spectrometers. Elemental analyses were performed by a CE Instruments EA1110 elemental analyzer. An Orion 3-Star pH meter with a glass electrode was used for the measurements of pH values after calibration with standard buffer solutions. ESI-MS data were collected on a Shimadzu LCMS-2020. Formate concentrations were monitored by an HPLC on an anion-exclusion column (Tosoh TSKgel SCX(H^+)) using an aqueous H_3PO_4 solution (20 mM) as an eluent and a UV detector (λ = 210 nm). Water used in the reactions was obtained from a Simplicity water purification system. Unless specifically noted, all reagents were purchased commercially without further purification. [Cp*Ir(OH_2)$_3$][SO_4] was synthesized according to a previous report.[1]

General Procedure for FA Dehydrogenation: A freshly prepared 10 mM aqueous solution of catalyst (100 μL) was added to a deaerated aqueous HCO_2H (FA)/HCO_2Na (SF) solution, and the mixture was stirred at the desired temperature. The volume of released gases was determined by a wet gas meter. The TOF was determined according to the released gases. The average TOF of the initial 10 min was adopted for the initial TOF. After the reaction was completed, the residual FA in the solution was quantified with HPLC. The TON was calculated based on the catalyst loading and concentration of residual FA or formate.

Note: The solubility of catalyst **2** in neutral water is relatively poor. Therefore, catalyst **2** was added to a FA solution after being dissolved in an alkaline solution with NaOH (pH > 12).

2. Synthesis of ligands.

2.1 Synthesis of **L1**.

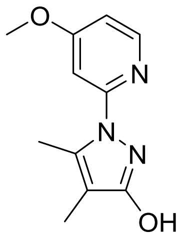

The mixture of 2-Bromo-4-methoxy-pyridine (3.0 g, 16 mmol), 3-hydroxy-4,5-dimethyl pyrazole (1.6 g, 14 mol), CuI (0.27 g, 1.4 mmol), K_2CO_3 (4.0 g, 29 mmol), and L-proline (0.34 g, 2.9 mmol) in DMSO (20 mL) was stirred under Ar for 48 h at 75 ^{o}C. After cooling to room temperature, water (100 mL) was added. The resulting mixture was extracted by EtOAc (30 mL×5). The combined organic phase was washed with water and brine (30 mL×3), dried over anhydrous $MgSO_4$, and concentrated to yield the crude product as a brown solid. The crude product was washed with MeOH (10 mL) and ^{i}PrOH (10 mL) to provide the product (540 mg, 2.5 mmol, yield: 17%) as a white solid. ^{1}H NMR (DMSO, 400 MHz): δ 10.23 (s, 1H), 8.17 (d, *J* = 5.8 Hz, 1H), 7.15 (d, *J* = 2.4 Hz, 1H), 6.78 (dd, *J* = 5.8 Hz, 2.4 Hz, 1H), 3.85 (s, 3H), 2.51 (s, 3H), 1.83 (s, 3H).

2.2. Synthesis of **L2**.

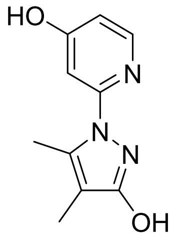

The mixture of **L1** (150 mg, 0.68 mmol) and 47% HBr (1.2 mL) in CH_3COOH (1.5 mL), was stirred for 9 h at 140 ^{o}C. After cooling to room temperature, the resulting precipitation was filtered. Purification was carried out by reprecipitation of aqueous 1M NaOH (10 mL) solution of the crude compound by addition of 0.5 M H_2SO_4 (10 mL) to obtain a white solid (120 mg, 86 %). ^{1}H NMR (DMSO, 400 MHz): δ 10.4 (br, 2H), 8.13 (d, *J* = 6.1 Hz, 1H), 7.08 (d, *J* = 2.2 Hz, 1H), 6.71 (dd, *J* = 6.1, 2.2 Hz, 1H), 2.46 (s, 3H), 1.85 (s, 3H).

2.3. Synthesis of **L3**.

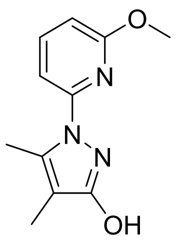

The mixture of 2-bromo-6-methoxy-pyridine (3.8 g, 20 mmol), 3-hydroxy-4,5-dimethyl pyrazole

(2.0 g, 18 mmol), CuI (0.35 g, 1.8 mmol), K_2CO_3 (5.1 g, 37 mmol), and L-proline (0.45 g, 3.9 mmol) in DMSO (20 mL) was stirred under Ar for 48 h at 75 °C. After cooling to room temperature, water (100 mL) was added. The resulting mixture was extracted with EtOAc (30 mL×5). The combined organic phase was washed with water and brine (30 mL×3), dried over anhydrous MgSO4, and concentrated to yield the crude product as a brown solid. The crude product was washed with ^{i}PrOH (10 mL) to yield the product (671 mg, 3.0 mmol, yield: 17%) as a white solid. ^{1}H NMR (DMSO, 400 MHz): δ 10.24 (s, 1H), 7.73 (dd, *J* = 6.3 Hz, 6.3 Hz, 1H), 7.19 (d, *J* = 6.3 Hz, 1H), 6.56 (d, *J* = 6.3, 1H), 3.86 (s, 3H), 2.57 (s, 3H), 1.84 (s, 3H).

2.4. Synthesis of **L4**.

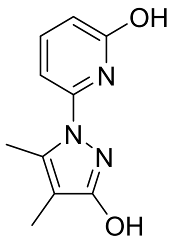

The mixture of **L3** (150 mg) and 47% HBr (1.2 mL) in CH_3COOH (1.5 mL), was stirred for 9 h at 140 °C. After cooling to room temperature, the resulting precipitation was filtered. Purification was carried out by reprecipitation of aqueous 1M NaOH (10 mL) solution of the crude compound by addition of 0.5 M H_2SO_4 (10 mL) to obtain a white solid (85 mg, 60 %). ^{1}H NMR (DMSO, 400 MHz): δ 10.6 (s, 1H), 10.2 (s, 1H), 7.66 (dd, *J* = 6.3 Hz, 6.3 Hz, 1H), 7.03 (d, *J* = 6.3 Hz, 1H), 6.39 (d, *J* = 6.3 Hz, 1H), 3.89 (t, *J* = 10.0 Hz, 2H), 2.51 (s, 3H), 1.82 (s, 3H).

3. Synthesis of complexes 1-5.

3.1. Synthesis of complex **1**.

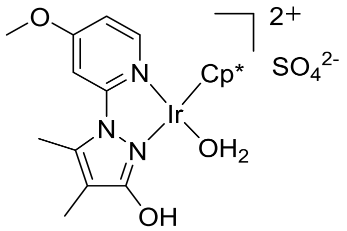

A mixture of $[Cp^*Ir(OH_2)_3]SO_4$ (324 mg, 0.68 mmol) and **L1** (150 mg, 0.68 mmol) in 20 mL H_2O was stirred at room temperature under Ar atmosphere for 18 h. The solution was filtered to remove the insoluble solid. The filtrate was concentrated and dried at 50 °C under vacuum for 5 h to yield the product as a yellow solid (440 mg, 97 %). ^{1}H NMR (D_2O, 400 MHz): δ 8.47 (d, *J* = 6.8 Hz, 1H), 7.11 (d, *J* = 2.5 Hz, 1H), 6.90 (dd, *J* = 6.8 Hz, 2.4 Hz, 1H), 3.92 (s, 3H), 2.53 (s, 3H), 1.87 (s, 3H), 1.59 (s, 15H). ^{13}C{^{1}H} NMR (D_2O, 100 MHz): δ 170.11, 164.27, 151.60, 151.19, 143.36, 109.96, 108.56, 98.03, 89.06, 57.17, 12.66, 8.80, 6.71. ESI-MS(m/z): $[M-H_2O-SO_4^{2-}-H^+]^+$ calcd for $C_{21}H_{27}IrN_3O_2^+$, 546.2; found, 546. Elemental analysis Calc. for

$C_{21}H_{30}IrN_3O_7S + 1.5H_2O$: C 36.67, H 4.84, N 6.11. Found: C 36.42, H 4.79, N 6.29.

3.2 Synthesis of complex **2**

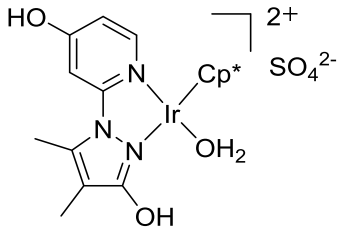

A mixture of $[Cp^*Ir(OH_2)_3]SO_4$ (237 mg, 0.50 mmol) and **L2** (100.0 mg, 0.49 mmol) in 20 mL H_2O was stirred at room temperature under Ar atmosphere for 18 h. The resulting precipitation was filtered. Purification was carried out by reprecipitation of aqueous 1M NaOH (10 mL) solution of the crude complex by addition of 0.5 M H_2SO_4 (10 mL) to obtain an orange solid (190 mg, 60 %). ^1H NMR (D_2O/NaOD, 400 MHz): δ 7.90 (d, J = 6.8 Hz, 1H), 6.50 (d, J = 2.4 Hz, 1H), 6.19 (dd, J = 6.8 Hz, 2.4 Hz, 1H), 2.39 (s, 3H), 1.73 (s, 3H), 1.51 (s, 15 H). $^{13}C\{^1H\}$ NMR (D_2O/NaOD, 100 MHz): δ 177.15, 169.53, 151.42, 149.20, 139.45, 114.45, 109.04, 99.39, 85.28, 12.96, 8.70, 7.02. ESI-MS(m/z): $[M{-}H_2O{-}SO_4^{2-}{-}H^+]^+$ calcd for $C_{20}H_{25}IrN_3O_2^+$, 532.2; found, 532. Elemental analysis Calc. for $C_{20}H_{28}IrN_3O_7S$: C 37.14, H 4.36, N 6.50. Found: C 37.17, H 4.24, N 6.63.

3.3. Synthesis of complex **3**.

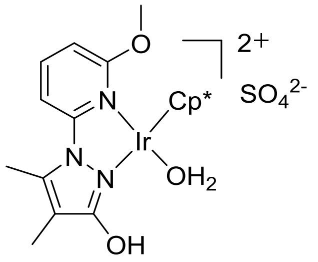

A mixture of $[Cp^*Ir(OH_2)_3]SO_4$ (324 mg, 0.68 mmol) and **L3** (150 mg, 0.68 mmol) in 20 mL H_2O was stirred at room temperature under Ar atmosphere for 18 h. The solution was filtered to remove the insoluble solid. The filtrate was concentrated and dried at 50 °C under vacuum for 5 h to yield the product as a yellow solid (423 mg, 94 %). ^1H NMR (D_2O, 500 MHz): δ 7.99 (dd, J = 8.4 Hz, 8.4 Hz, 1H), 7.33 (d, J = 8.4 Hz, 1H), 6.90 (d, J = 8.4 Hz, 1H), 4.09 (s, 3H), 2.57 (s, 3H), 1.90 (s, 3H), 2.53 (s, 15H). $^{13}C\{^1H\}$ NMR (D_2O, 125 MHz): δ 164.86, 163.51, 148.88, 145.88, 144.60, 108.53, 104.17, 103.57, 89.26, 58.24, 12.77, 9.30, 6.71. ESI-MS(m/z): $[M{-}H_2O{-}SO_4^{2-}{-}H^+]^+$ calcd for $C_{21}H_{27}IrN_3O_2^+$, 546.2; found, 546. Elemental analysis Calc. for $C_{21}H_{30}IrN_3O_7S + H_2O$: C 37.17, H 4.75, N 6.19. Found: C 36.95, H 4.52, N 6.00.

3.4. Synthesis of complex [Cp*Ir(**L4**)(OH_2)]SO_4 (**4**).

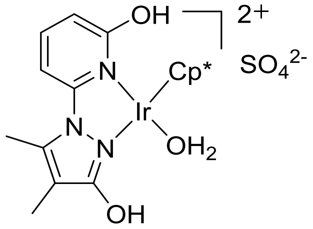

A mixture of [Cp*Ir(OH_2)$_3$]SO_4 (160 mg, 0.34 mmol) and **L4** (70 mg, 0.30 mmol) in 10 mL H_2O was stirred at room temperature under Ar atmosphere for 18 h. The solution was filtered to remove the insoluble solid. The filtrate was concentrated and dried at 50 °C under vacuum for 5 h to yield the product as a yellow solid (218 mg, 97 %). ^{1}H NMR (D_2O, 400 MHz): δ 7.80 (dd, *J* = 8.0 Hz, 8.0 Hz, 1H), 7.16 (d, *J* = 8.0 Hz, 1H), 6.73 (d, *J* = 8.0 Hz, 1H), 2.54 (s, 3H), 1.89 (s, 3H), 1.53 (s, 15H). ^{13}C{^{1}H} NMR (D_2O, 100 MHz): δ 163.94, 163.27, 148.37, 144.96, 144.32, 107.80, 107.27, 102.41, 89.22, 12.70, 9.35, 6.65. ESI-MS(m/z): [M−H_2O−SO_4^{2-}−H^+]$^+$ calcd for $C_{21}H_{27}IrN_3O_2^+$, 532.2; found, 532. Elemental analysis Calc. for $C_{20}H_{28}IrN_3O_7S$: C 37.14, H 4.36, N 6.50. Found: C 36.99, H 4.37, N 6.58.

3.5. Synthesis of complex **5**.

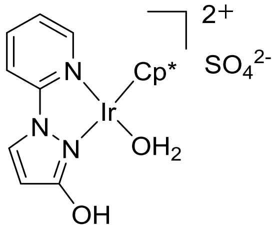

A mixture of [Cp*Ir(OH_2)$_3$]SO_4 (205 mg, 0.43 mmol) and 3-hydroxy-1-(2-pyridyl)-1H-pyrazole (70 mg, 0.43 mmol) in 20 mL H_2O was stirred at room temperature under Ar atmosphere for 18 h. The solution was filtered to remove the insoluble solid. The filtrate was concentrated and dried at 50 °C under vacuum for 5 h to yield the product as a yellow solid (235 mg, 91 %). ^{1}H NMR (D_2O, 400 MHz): δ 8.71, 8.37, 8.13, 7.81, 7.44, 6.26, 1.64. ^{13}C{^{1}H} NMR (D_2O, 100 MHz): δ 165.51, 149.36, 149.25, 143.04, 133.08, 123.63, 110.89, 99.32, 89.14, 8.44. Elemental analysis Calc. for $C_{18}H_{24}IrN_3O_6S$ + H_2O: C 34.83, H 4.22, N 6.77. Found: C 34.93, H 4.18, N 6.91.

Figure and Table

Table S1. The results of FADH under various temperature conditions.[a]

Cat.	Temp, °C	1/T, K	TOF[b], h^{-1}	ln(TOF)
**1**	50	0.003096	3700	8.22
**1**	60	0.003003	8210	9.01
**1**	70	0.002916	16,700	9.72
**1**	80	0.002833	36,000	10.49
**2**	50	0.003096	4150	8.33
**2**	60	0.003003	8710	9.07
**2**	70	0.002916	18,800	9.84
**2**	80	0.002833	39,850	10.59

[a] Reaction conditions: 10 mL 1 M FA solution or FA, 1 μmol catalyst, [b] Average TOF over the initial 10 min.

Table S2. FA dehydrogenation in the presence of impurities.[a]

Entry	Impurity	Time, h	TOF, h^{-1}
1	None	4	8100
2	2000 eq. MeOH	4	7750
3	200 eq. Na_2SO_4	4	8400
4	2000 eq. Na_2SO_4	4	7890
5	100 equiv. CH_3COOH	4	7750
6	20 equiv. NaCl	6	4180
7	100 equiv. NaCl	11	1970

[a] The reaction was carried out in deaerated aqueous FA solution. Conditions: [FA] = 1.0 M, [cat] = 50 μM, at 60 °C. [b] The initial TOF was measured after 10 min.

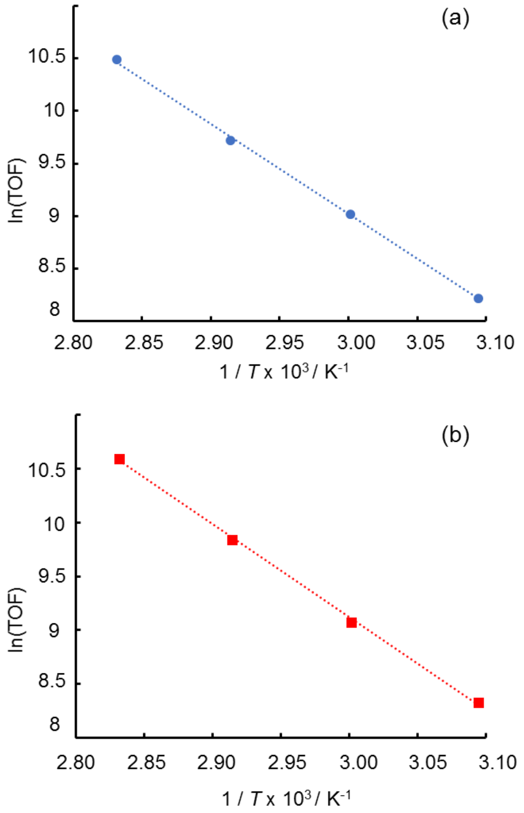

Fig. S1. Arrhenius plots for FADH (a) with **1** and (b) with **2**. Reaction conditions: [FA] = 1.0 M, [cat] = 100 μM.

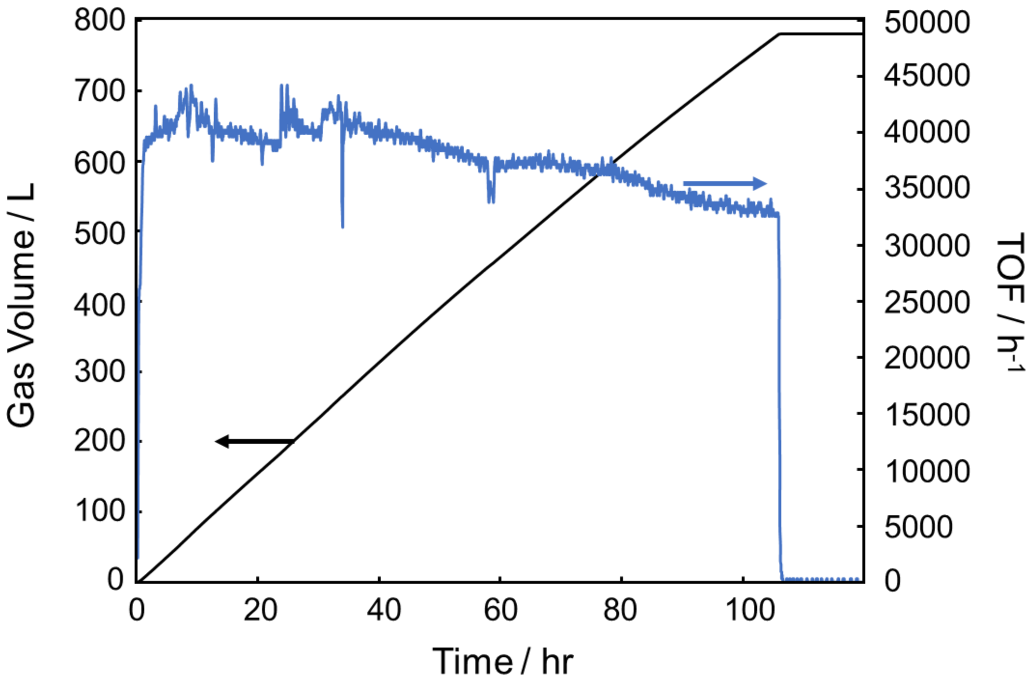

Fig. S2. Time courses of volume of released gases and rate of released gases in FADH with the continuous addition of neat FA by a pump. Conditions: $[FA]_0$ = 8 M, 50 mL, $[\mathbf{1}]_0$ = 80 μM, 70 °C, rate of neat FA addition = 0.1 ml/min.

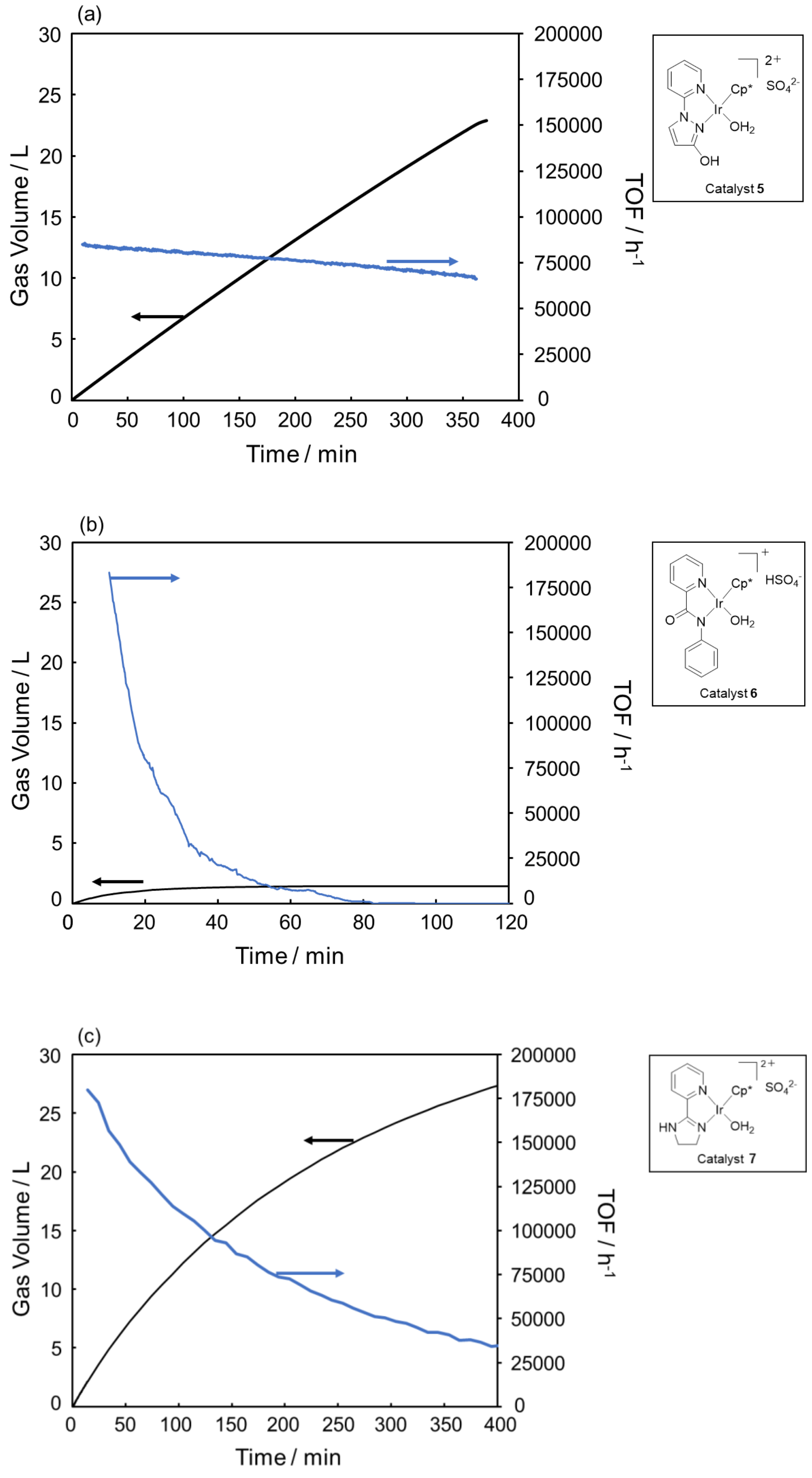

Fig. S3. Time courses of volume of released gases and rate of released gases in FADH catalyzed by catalyst **5-7**. Conditions: [FA] = 8 M, 100 mL, [**cat**] = 10 μM, reflux).

Reference:

1. Ogo, S.; Makihara, N.; Watanabe, Y. *Organometallics* **1999**, *18*, 5470-5474.
